# ABO Blood Group Is Associated with Response to Inhaled Nitric Oxide in Neonates with Respiratory Failure

**DOI:** 10.1371/journal.pone.0045164

**Published:** 2012-09-12

**Authors:** George T. El-Ferzli, Mackenzie Dreher, Rakesh P. Patel, Namasivayam Ambalavanan

**Affiliations:** 1 Departments of Pediatrics, Centers for Free Radical Biology and Pulmonary Injury Repair Center, University of Alabama at Birmingham, Birmingham, Alabama, United States of America; 2 Departments of Pathology, Centers for Free Radical Biology and Pulmonary Injury Repair Center, University of Alabama at Birmingham, Birmingham, Alabama, United States of America; Emory University School of Medicine, United States of America

## Abstract

**Background:**

Inhaled nitric oxide (iNO) reduces death or need for extracorporeal membrane oxygenation (ECMO) in infants with persistent pulmonary hypertension of the newborn (PPHN). However, the response to iNO is variable and only 50–60% of infants demonstrate a response to iNO. It is not known why only some infants respond to iNO. Adults and children with blood groups B or AB do not respond as well to iNO as those with blood groups O/A.

**Methods/Principal Findings:**

To determine if blood group was associated with iNO response in newborn infants, a retrospective medical record review was done of infants admitted to a regional NICU from 2002-9 with a diagnosis of PPHN. Data were collected during the first twelve hours post-initiation of treatment. Of 86 infants diagnosed with PPHN, 23 infants had blood group A [18 received iNO], 21 had group B [18 with iNO], 40 had group O [36 with iNO], and 2 had group AB [both received iNO]. Change in PaO_2_/FiO_2_ was less in infants with blood group A, of whom less than half were responders (ΔPaO_2_/FiO_2_>20%) at 12 h versus 90% of infants with either O or B. Race, sex, birth weight, gestational age, Apgar scores at 1 and 5 minutes, and baseline PaO_2_/FiO_2_ were similar among groups. Outcomes including need for ECMO, death, length of ventilatory support, length of iNO use, and hospital stay were statistically not different by blood groups.

**Conclusions/Significance:**

Our results indicate that blood group influences iNO response in neonates. We hypothesize that either there is genetic linkage of the ABO gene locus with vasoregulatory genes, or that blood group antigens directly affect vascular reactivity.

## Introduction

Inhaled nitric oxide (iNO) improves oxygenation and reduces need for extracorporeal membrane oxygenation in term and near-term infants with respiratory failure [1]. However, improvement in oxygenation does not occur in all infants, and the percentage of infants responding to iNO has ranged from 50–60% in randomized controlled trials [2]–[5]. It is not understood why some infants respond to iNO while others do not improve in oxygenation, although differences in clinical management may be contributory. A randomized trial by Kinsella et al.[6] comparing iNO with conventional ventilation to high frequency oscillatory ventilation (HFOV) without iNO, with treatment failures managed with HFOV+iNO, suggested that a failure to achieve optimal lung expansion may lead to inadequate response to iNO. However, even under the carefully controlled conditions of this trial, 40% of the infants did not respond to either HFOV or iNO or the combination of HFOV+iNO [6]. Weimann and colleagues have shown that adults with acute respiratory distress syndrome and blood groups B/AB did not respond as well to iNO as those with blood groups O/A [7]. McFadzean and colleagues had observed in children that although there were no differences in proportion of responders (defined as >20% improvement in PaO_2_/FiO_2_ within 6 h) by blood group, responders with group B/AB took longer to improve in PaO_2_/FiO_2_ compared to group O/A [8]. To determine if a similar association of blood group with response to iNO was present in newborn infants, we performed a retrospective chart review. Infants admitted over an eight year period (2002-9) to the regional neonatal intensive care unit at UAB with a clinical and echocardiographic diagnosis of persistent pulmonary hypertension of the newborn (PPHN) were included. Infants with congenital heart disorders were excluded from analysis. Data were collected for the time period one hour before initiation of iNO to 48 h later. The PaO_2_ was evaluated at baseline, 1 hour, 6 hours, 12 hours, 24 and 48 hours after initiation of iNO. This study was approved by the Institutional Review Board of the University of Alabama at Birmingham (UAB), with waiver of informed consent, and data were analyzed anonymously.

## Results

**Figure 1:**
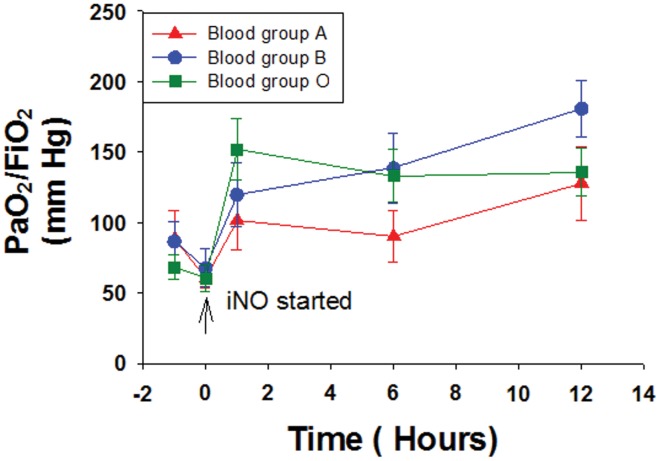
Magnitude of response in PaO_2_/FiO_2_ in relation to blood group at various time points before and after initiation of iNO. (mean±SE; n = 18 for group A, 18 for group B, and 36 for group O). By Repeated Measures ANOVA, p<0.01 for blood groups B and O, and p = 0.18 for blood group A for changes in PaO_2_/FiO_2_ over time.

Of 86 infants diagnosed with PPHN, 23 (27%) were blood group A, 21 (24%) group B, 40 (47%) group O, and 2 (2%) group AB. Of these infants, 18 with group A (78%), 18 with group B (82%), and 36 of group O (90%) received iNO. The two infants with group AB were excluded from further analysis due to the small sample size. Baseline characteristics of the infants including birth weight (3466±122 g for group A, 3017±190 g for group B, 3387±88 g for group O), gestational age (median 38 weeks), Apgar scores at 1 (median 6) and 5 minutes (median 7), and initial PaO_2_/FiO_2_ (Table 1, Figure 1) were similar among groups. When a ΔPaO_2_/FiO_2_ of >20% at 12 hours after initiation was defined as a response, there were more responders over time in blood group B or O (**Type A**: 39% at 1 h, 50% at 6 h, 44% at 12 h; **Type B**: 50% at 1 h, 75% at 6 h, 92% at 12 h; **Type O**: 61% at 1 h, 71% at 6 h, 89% at 12 h; p<0·01 by z-test for proportions between Type A vs. Types B and O at 1 h and 12 h). Therefore, less than half of group A infants were responders at the 12 h time point as compared to approximately 90% of infants with either groups O or B. However, there was marked within-group variability at all time points in absolute PaO_2_/FiO_2_ and delta PaO_2_/FiO_2_ (Figure 1). When changes over time in PaO_2_/FiO_2_ were evaluated by repeated measures ANOVA in the different blood groups, infants with group A did not show a significant change over time (p = 0.12), while PaO_2_/FiO_2_ in infants with group B (p = 0.01) and group O (p<0.001) were significantly different from baseline. However, when results were analyzed by two-way ANOVA (PaO_2_/FiO_2_ in relation to the two factors of blood group and time of blood gas), the effect of time on PaO_2_/FiO_2_ remained significant (p = 0.002), but the differences noted for blood group were no longer significant (p = 0.29) likely due to variability and small sample size.

**Table 1:** Characteristics and Outcomes of Infants by Blood Group.

Blood Group	A (n = 23)	B (n = 21)	O (n = 40)
**Gender: Male**/**Female**	14/9	12/9	25/15
**Race: White/Black**/**Hispanic**	13/4/3	7/12/1	19/15/6
**Birth Weight (g) (mean ± SEM)**	3466**±**122	3017**±**190	3387**±**88
**Gestational Age in Weeks (Median)**	38	38	39
**Apgar - 1 minute (Median)**	6	5	6
**Apgar - 5 minutes (Median)**	8	7	7
**Age at admission in days (Median)**	2	2	2
**Age iNO started in days (Median)**	2.2**±**0.2	2.3**±**0.4	2.8**±**0.3
**iNO length in days (Median)**	3.7**±**0.7	4**±**0.7	4.2**±**0.6
**Survival (%)**	20/23 (87%)	19/21 (90%)	38/40 (95%)
**Length of Stay in days (mean ± SEM)**	14.6**±**2.0	14.2**±**1.8	12.9**±**1.8
**Duration of mechanical ventilation in days (mean ± SEM)**	7.5**±**1.0	7.3**±**1.0	7.3**±**1.3

Outcomes including need for ECMO (5% for groups A and B, 14% for group O), death (13% with group A, 10% with group B, 9% with group O), length of ventilatory support (mean 7.3 to 7.5 days in all groups), length of iNO use (3.7±0.7d with group A, 4±0.7d with group B, 4.2±0.6 with group O), and length of hospital stay (15±2 d with group A, 14±2d with group B, 13±2d with group O) were not statistically different (all p>0.05) between blood groups (Table 1). No differences in pH and PaCO_2_ were noted between the groups at baseline or at other time points (data not shown).

By backward stepwise regression, lower birth weight, earlier gestational age, lower 5 minute Apgar score, blood group A, and longer ventilator support were predictive of death (p<0.05); the magnitude of response in PaO_2_, either as a continuous or categorical variable, could not be modeled using the available variables. There was no gender effect with respect to the response to iNO.

## Discussion

Our study confirmed that the response in terms of oxygenation to iNO is variable in neonates with PPHN. Our results indicate that blood group is associated with the oxygenation response to iNO in newborn infants with PPHN, and may account for some of the variability in response to iNO. The infants with blood type A had the least improvement in PaO_2_ in response to iNO.

Our results are in the opposite direction from those of Weimann et al.[7] and McFadzean et al.[8] who observed that blood group B had worse response to iNO. We suggest two alternative hypotheses for this phenomenon. One hypothesis is that there is genetic linkage of the *ABO* gene locus on chromosome 9q34 with other genes regulating vasoconstriction or vasodilation (e.g. dopamine beta hydroxylase [*DBH*], a voltage-dependent calcium channel subunit [*CACNA1B*], and prostaglandin D2 synthase [*PTGDS*], along with many other genes are all present on 9q34) or with genes regulating vascular events (e.g. increased vWF and Factor VIII have been described in blood groups A and B versus O)[9], [10] and these factors are developmentally regulated, such that the fetal/neonatal transitional circulation responds differently from that of the older child/adult. Previous studies have identified *ABO* as a locus for inflammatory biomarkers E-selectin[11], P-selectin[12], and soluble ICAM1[13], low-density lipoprotein (LDL-C) [14], and angiotensin-converting enzyme[15], which may all have a role in PPHN. The other hypothesis is that there is direct association of the blood group antigen with vascular reactivity and response to nitric oxide. It is known that ABO antigens are expressed on many epithelial and endothelial cells in addition to red blood cells (and hence blood groups are considered in transplantation of solid organs), and these antigens may have multiple functions that affect vascular tone (e.g. ion channels, transporters)[16] and are developmentally regulated. These hypotheses may be tested by identifying if the specific nature of the ABO-blood group isoantigen on pulmonary vascular smooth muscle or endothelial cells affects their ability to vasodilate blood vessels in response to iNO or other vasodilators, and if these effects are different in the fetus/neonate as compared to the adult. These studies may be clinically relevant and physiologically important as they may indicate potential therapeutic strategies in vasomodulation.

A limitation of our study is the relatively small sample size. A larger sample size would enable the determination of the independent contribution of blood group to oxygenation response. Another limitation is that the genotype (e.g. AA or AO for infants with group A, or A1 and A2 subtype) information was not available, and it is possible that potential differences between responders and non-responders may be due to differences in genotype.

## Materials and Methods

Infants admitted with persistent pulmonary hypertension of the newborn (PPHN) to a regional neonatal intensive care units (the University of Alabama at Birmingham) from 2002–2009 were evaluated. Infants were included in this study if the clinical diagnosis of PPHN was confirmed on echocardiography. Infants with congenital heart disease were excluded. Arterial blood gas data were collected, at time points prior to iNO initiation and at 0, 1, 6, 12, 24 and 48 hours after beginning iNO. It was usual clinical practice to initiate iNO at 20 ppm, with slow weaning over subsequent days. A “response” to iNO was defined as a change in ΔPaO_2_/FiO_2_ of >20% at 12 hours after baseline arterial blood gas (t = 0; just before iNO was started). The time point of 12 hours was chosen as we felt that this time point may be more clinically relevant as it would indicate a more sustained response, as compared to a response at 1 hour or 6 hours after initiation. Standard univariate and multivariate statistical analysis was done.
